# Can muscle vibration be the future in the treatment of cerebral palsy-related drooling? A feasibility study.

**DOI:** 10.7150/ijms.34850

**Published:** 2019-09-20

**Authors:** Emanuele F. Russo, Rocco S. Calabrò, Patrizio Sale, Filomena Vergura, Maria C. De Cola, Angela Militi, Placido Bramanti, Simona Portaro, Serena Filoni

**Affiliations:** 1 Padre Pio Foundation and Rehabilitation Centers, San Giovanni Rotondo, Foggia, Italy;; 2 IRCCS Centro Neurolesi Bonino-Pulejo, Messina, Italy; cristina.decola@gmail.com; 3 Rehabilitation Unit, Department of Neurosciences, University of Padua;; 4 Dipartimento di Scienze Biomediche odontoiatriche e delle immagini Morfologiche e Funzionali, University of Messina, Italy

**Keywords:** Muscle vibration, neurorehabilitation, developmental disorders, sialorrhea.

## Abstract

**Background:** Drooling is an involuntary loss of saliva from the mouth, and it is a common problem for children with cerebral palsy (CP). The treatment may be pharmacological, surgical, or speech-related. Repeated Muscle Vibration (rMV) is a proprioceptive impulse that activates fibers Ia reaching the somatosensory and motor cortex. **Aim:** The aim of the study is to evaluate the effectiveness of rMV in the treatment of drooling in CP. **Design, setting and population:** This was a rater blinded prospective feasibility study, performed at the “Gli Angeli di Padre Pio” Foundation, Rehabilitation Centers (Foggia, Italy), involving twenty-two CP patients affected by drooling (aged 5-15, mean 9,28 ± 3,62). Children were evaluated at baseline (T0), 10 days (T1), 1 month (T2) and 3 months (T3) after the treatment. **Methods:** The degree and impact of drooling was assessed by using the Drooling Impact Scale (DIS), the Drooling Frequency and Severity Scale (DFSS), Visual Analogue Scale (VAS) and Drooling Quotient (DQ). An rMV stimulus under the chin symphysis was applied with a 30 min protocol for 3 consecutive days. **Results:** The statistical analysis shows that DIS, DFSS, VAS, DQ improved with significant differences in the multiple comparisons between T1 vs T2, T1 vs T3 and T1 vs T4 (p≤0.001). **Conclusion** This study demonstrates that rMV might be a safe and effective tool in reducing drooling in patients with CP. The vibrations can improve the swallowing mechanisms and favor the acquisition of the maturity of the oral motor control in children with CP.

## Introduction

The widespread incidence of Cerebral palsy (CP) in childhood is 1-5 per 1000 live births 1, and it is the most frequent motor disability during this period. CP is considered a neurological disorder caused by a non-progressive brain injury or malformation that occurs while the child's brain is under development. The disease primarily affects body movement and muscle coordination, but may determine intellectual disabilities and behavioral abnormalities. Sometimes there could be epilepsy and secondary musculoskeletal problems 2.

Saliva has a fundamental role in keeping humid the mouth and preserving oral hygiene, making the bolus smooth while swallowing and regulating esophageal acidity. The submandibular glands (70%) produce the majority of saliva and only 30% is produced by the other glands 3.

The incapacity of controlling saliva in the mouth is due to poor head and lip control and/or tongue incoordination with a mouth constantly open or an diminished tactile sensation. Other causes can be macroglossia, nasal obstruction or dental malocclusion 4.

The incapacity to tackle oral secretions caused by oro-motor disorders is termed drooling or sialorrhea. Until the age of 18 months, drooling is normal, and it is accepted until the age of four. 5-7. Drooling may affect up to 45 % of CP patients, and can be classified into anterior and posterior. The former is clinically visible and it occurs in the oral phase of swallowing, whilst the latter is concerned with the spilling of saliva on the tongue due to the facial isthmus, and it regards the pharyngeal phase in patients with serious oropharyngeal dysphagia 5-8.

Drooling can cause distress and affliction not only in children, but also in parents and caregivers, due to bad smelling, irritated or macerated facial skin, orofacial infections, dehydration, speech and masticatory problems 9. The probability of aspiration pneumonia and chest infections are higher. Unfortunately, all these problems can lead to social isolation. 9. The various available treatments include anticholinergic drugs, rehabilitation, kinesio-taping, botulinum toxin injection and, in specific cases, surgery 10-13.

Some studies have assessed the validity of rehabilitation by using oro-motor therapy, behavioral approaches and biofeedback. The use of sublingual, oral and cutaneous medication (muscarinic cholinergic receptor antagonists) is now limited because there is very little evidence of its validity and it may have side effects 14. Although some studies have demonstrated the efficacy of botulinum toxin 12, the best approach to this devastating problem has not been determined yet 15-16.

The use of vibratory stimuli has demonstrated practical applications in the areas of therapeutic rehabilitation and exercise performance. Muscle vibration is a technique that applies a low-amplitude/high-frequency vibratory stimulus to a specific muscle using a mechanical device. Repeated Muscle Vibration (rMV) is a proprioceptive impulse that activate fibers Ia reaching the somatosensory and motor cortex. rMV has been employed in rehabilitation in many cases with considerable results. It has been demonstrated that rMV may reduce spasticity 17, and facilitate motor control tasks 18, improve fatigue resistance, time of force development and strength 19, intensify muscle contraction 20, and improve gait 21.

The aim of the study is to evaluate a new technique based on rMV for the treatment of drooling in patients with CP. We postulated that rMV might improve drooling by boosting oral motor control, considering its positive effects on muscle coordination and strength.

## Materials and methods

### Study design and population

This was a rater blinded prospective pilot study performed at the “Gli Angeli di Padre Pio” Foundation, Rehabilitation Centers, Foggia, Italy. Among the 50 CP patients screened for study enrolment, twenty-two children met the inclusion criteria and entered the study. The children (8 males, 14 females, aged 5 - 15 years, mean 9,28 ± 3,62) were enrolled from February 2016 to April 2018.

Inclusion criteria were: i) confirmed diagnosis of cerebral palsy, ii) score of ≥ 6 on DFSS, iii) age between 5 and 18 years, and iv) informed consent obtained by the parents/caregivers. Exclusion criteria were: i) previous surgical interventions for saliva control, ii) use of drugs that could interfere with saliva secretion (including botulinum toxin) and iii) involvement in other medical studies.

This study was approved by the Local ethics committee (IRCCSME-ID 29/2015), and was performed in accordance with the Declaration of Helsinki. All parents or caregivers gave their informed consent for this study.

**Fig 1:**
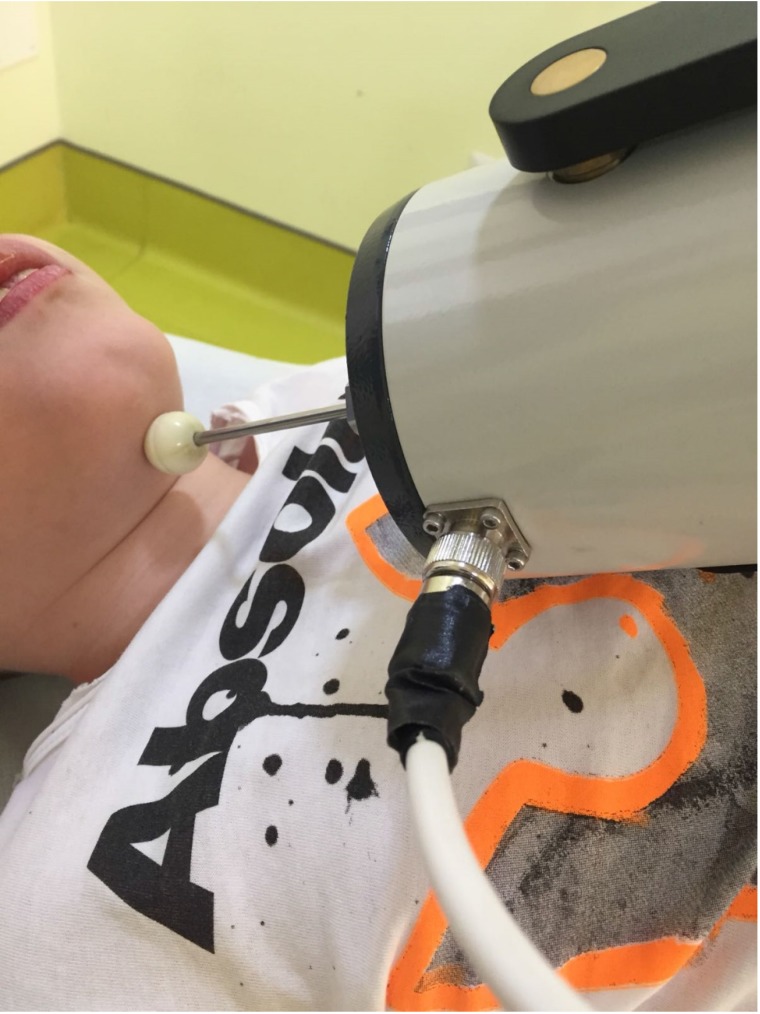
shows the transducer position during drooling treatment in a patient with cerebral palsy.

Muscle vibration was applied by means of the Cro®System (Pacioni&C. S.n.c, Italy), an electromechanical transducer with a particular mechanical support. We used a low amplitude rMV at a fixed frequency of 100 Hz. Thanks to a little probe (diameter of 10mm), the vibration was located over submandibular muscles, behind mandibular symphysis, i.e. digastric, mylohoyid, hyoglossus, geniohyoid, genioglossus and styloglossus muscles (Fig. 1). The transducer was directed so that it produced sinusoidally modulated forces ranging between 7 and 9 N. The range of vibration amplitude was from 0.05 to 0.1mm.

The training lasted 3 consecutive days, and was performed three times a day. Every application lasted 10 minutes and there was an interval of 60s between the three applications, so that the person's muscles could relax.

### Outcome Measures

Children were evaluated by a skilled speech therapist, at baseline (T0), 10 days (T1), 1 month (T2) and 3 months (T3) after the treatment.The degree and impact of drooling was assessed by means of the Drooling Impact Scale (DIS) and with the Drooling Frequency and Severity Scale (DFSS), Visual Analogue Scale (VAS), Drooling Quotient (DQ). Every measurement was performed in the morning under normal conditions about 1 hour after mealtime.

The DQ (expressed as a percentage) is a method, which is semi-quantitative obtained by observation. After having wiped off the saliva from the chin and any trace of food that had remained in the mouth was taken away too, the drooling quotient assessment started. DQ was recorded, registering the episodes of drooling that took place during two stages of 5 minutes that were separated by an interval of 30 minutes 22. When new saliva appeared on the lip margin or drooling started from the chin, it was considered as an episode of drooling. Every 15 seconds, for 5 minutes (totally 20 assessments) there was a control to verify if drooling was occurring or not. During the DQ 'rest' condition, the child could watch TV, sit in an upright position on his wheelchair, but he did not have to talk. In the DQ “activity” condition, according to the child's interests and his abilities they could perform different activities such as using electronic communication devices or play building blocks.

In the DIS questionnaire that we distributed the week before, there were 10 questions rated from 1 to 10 on a semantic differential scale 23. The total scoring of the questionnaire gives a general evaluation on the impact that drooling has on the child and the severity of drooling. The maximum possible total for the scale was 100. To evaluate the frequency of drooling and its severity the DFSS scale was adopted 24. Every person was attributed with a grade that corresponded to these definitions: 1, dry (when there was no drooling); 2, mild (when only the lips were wet); 3, moderate (when the lips and chin were wet); 4, severe (when drooling wetted clothes); 5, profuse (when it wetted clothes, hands and objects). The frequency of drooling was rated too: 1, no drooling; 2, sporadic drooling; 3, repeated drooling, 4, unceasing drooling. Taking into consideration the values of both scales, a combined drooling scale was formed that went from 2 to 9. In addition, the parents were administered a VAS scale, to get their impression on the symptom severity (0 absence of drooling, 100 = exaggerated drooling).

### Statistical analysis

The normality of the distribution of all variables was assessed by the Shapiro -Wilk statistic. Data are reported as Median and Interquartile Range (IQR). For every outcome variable taken into consideration, to prove the differences among the different assessment period, the Friedman test was adopted, after that Wilcoxon signed rank test and Holm-Bonferroni sequential correction were carried out for multiple comparisons 25.

For every analysis p values <0.05 were regarded significant in terms of statistics.

## Results

All participants completed the three sessions of the treatment, without reporting any significant adverse event. Table 1 summarizes the participants' characteristics.

**Fig 2:**
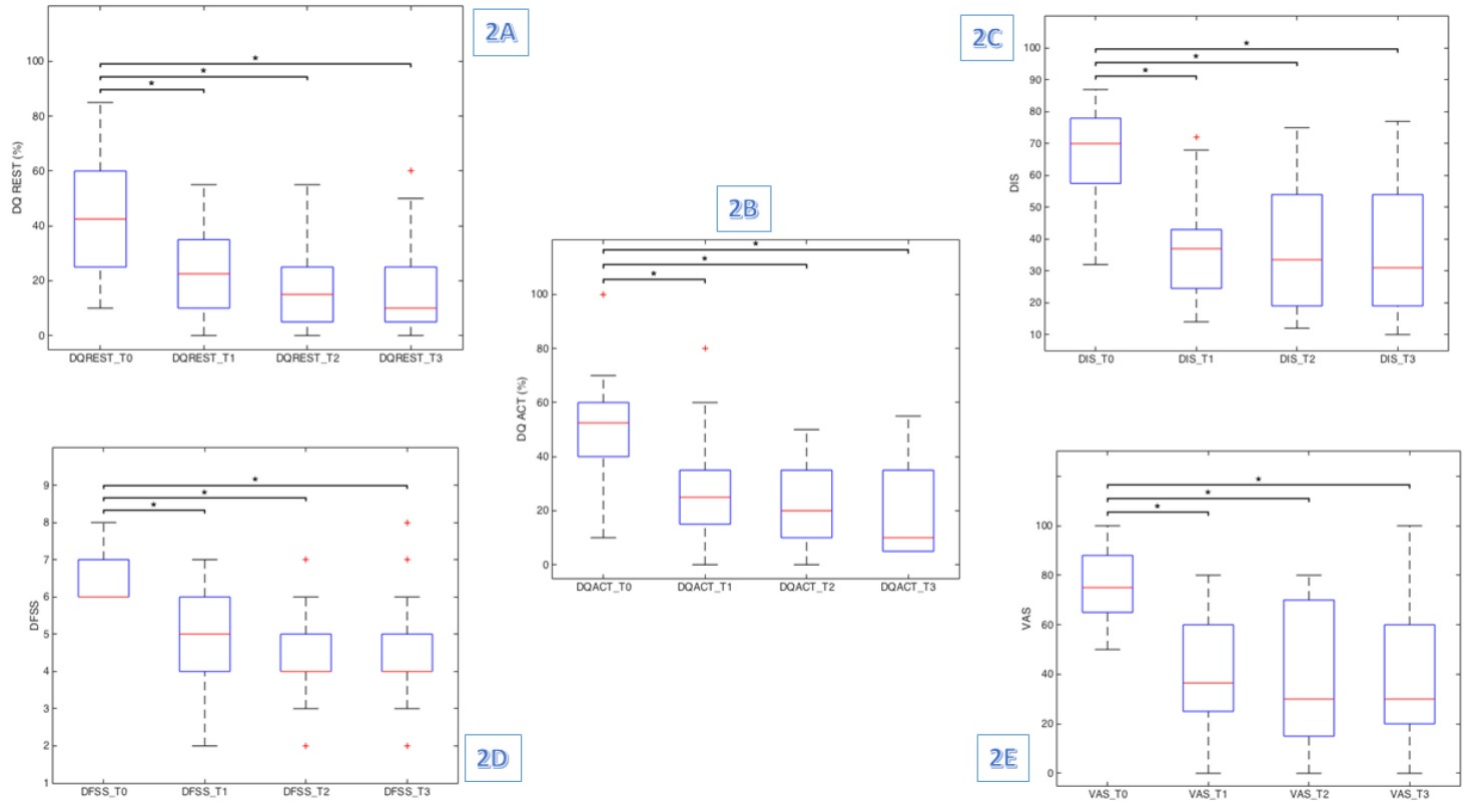
shows the box plot diagram for a) DQREST, b) DQACT, c) DIS, d) DFSS and e) VAS. * Wilcoxon signed rank test (Holm- Bonferroni sequential correction)

Friedman test results were significant for all clinical test scores administered, demonstrating a significant reduction among the assessment time points in DQREST (χ2 (3) = 29.099, p≤0.001), DQACT (χ2 (3) = 35.250, p≤0.001), DIS (χ2 (3) = 34.422, p≤0.001), VAS (χ2 (3) = 31.010, p≤0.001) and DFSS (χ2 (3) = 34.153, p≤0.001) scores. Indeed, we found a significant reduction in frequency, intensity and severity of drooling at rest and during patient's activities (Fig 2).

However, the post-hoc analysis revealed statistically significant differences only between baseline (T0) and the other assessment time points (T1, T2 and T3), as showed in Table 2. Hence, the score changes are significant from baseline to post-treatment and after the improvement remains stable (table 3).

## Discussion

To the best of our knowledge, this is the first attempt to evaluate the effect of focal muscle vibration in the treatment of sialorrhea. Our pilot study support our idea that rMV could be a valuable tool to improve drooling in children with CP. Indeed, we found a significant reduction in frequency, intensity and severity of drooling at rest and during patient's activities, as demonstrated by the clinical test administered before and after the treatment.

Besides the medical problems, this annoying symptom can be considered a social disability, as it becomes an obstacle for social interaction. Consequently, it has a negative impact on both CP patients and caregivers' quality of life, being drooling quite common in such neurological disorder. Although drooling may have different causes, in CP patients it is more due to a disturbed deglutition than to hypersalivation 26. Indeed, due to neurodevelopmental delay there could be a disturbance of some primary functions, including oral sensibility, swallowing, lip closure, and suction. However, in the presence of saliva overflow, the most probable cause is an incoordination of tongue mobility, as it has been demonstrated that the quantity of saliva produced remains constant. In CP patients an abnormal coordination of head and trunk, and orofacial and palatolingual musculature, should be also considered 27-29.

Thus, although many factors may contribute to drooling, the problem in CP depends mainly on the lack of oral motor control. Motor control and muscle strengthening can be influenced by a powerful proprioceptive stimulation, as rMV reaches undeviatingly both the SI and MI by activating (at low amplitudes) Ia afferent fibers. The straight connections between SI and MI cortices supplies the anatomical substrate necessary for the function played by MV in reorganizing the motor and somatosensory cortices 17.

This is the reason why, for the ever first time, we decided to use rMV for the treatment of drooling in these patients.

Although there are different therapeutic ways of treating sialorrhea, we believe that rMV could be of some help in improving the disabling symptom. Speech therapy training could be a good solution because it treats the causes and its long-term effects, but it depends entirely on the child's intellectual capacities and, besides, the treatment has to be repeated with regular frequency too 4. The rMV treatment, instead, has the advantage of being applied in shorter sessions and its validity depends only on the correct target-muscle positioning of the transducer.

Botulinum toxin is valid and safe treatment of sialorrhea, but it has many disadvantages 11,15,16. Indeed, it is only temporarily effective, as every 3-6 months the patient should repeat it, and it is very expensive due to both the drug costs and the need of highly qualified multidisciplinary staff 4, 11. Moreover, the main disadvantage of the treatment is that it concentrates only on the effect (i.e. reduction of saliva production) and not on the causes (i.e. oral motor incoordination), as instead rMV does 12, 26.

The surgical treatment, although definitive and valid, should be considered only in selected cases, as it needs a specialized team and general anesthesia 10. Finally, the use of specific drugs, such as anticholinergics, may lead to undesirable and harmful side effects, including insomnia, irritability, diarrhea and vomiting 14.

Differently from most of the previous studies, the rMV approach acts on the cause of drooling and not on the effects. rMV can also be used in non-collaborating individuals (as most of the patients with intellectual disability are) that would not allow them to actively participate in a speech therapy training, as those enrolled in our study.

Notably, we found that the improvement of drooling was already evident one week after the end of the treatment and lasted up to three months. The treatment was well tolerated and safe, there were no adverse events and dropouts.

We have applied focal vibrations under the chin symphysis to stimulate the muscles behind mandibular symphysis, i.e. digastric, mylohoyid, hyoglossus, geniohyoid, genioglossus and styloglossus muscles. This vibratory stimulus may therefore have affected the orofacial and lingual palate mechanisms, improving coordination, muscle tone, strengthening the muscles and giving a great sensorial stimulus.

Indeed, it is possible that the improvement of the coordination and strength mechanisms of the treated muscles could favor the improvement of swallowing through the acquisition of a maturity of the oral motor control. Consequently, the best management of saliva inside the mouth causes an immediate improvement of the drooling. Swallowing, once acquired, is constantly trained in the activities of daily life, thus probably potentiating the effects of the treatment, also at follow-up.

The rMV training could have boosted connectivity within the sensorimotor areas by activating Ia fibers 31, 32. Indeed, such higher vibration frequencies have been shown to elicitate motor response, spinal and supra spinal reflexes and the activation of suprasegmental structures so to modify motor command strategies. More in detail, it has been shown that the rMV-induced modifications are very likely due to at least 2 forms of plasticity: i) a form of nonsynaptic plasticity that induces changes in the intrinsic properties of neural membranes (explaining the lowering of the motor threshold), and ii) a Hebbian-like mechanism of synaptic plasticity, which may account for the functional restoration of inactivated, though preserved, motor pathways and/or rearrangements of motor cortical maps 17.

This is the reason why we may argue that the use of vibrations is an effective and potentially long-lasting method for treatment for sialorrhea.

The main limitations of this study include the low number of participants and the absence of a control group. However, this is a pilot study, and further larger sample randomized trials are needed to confirm these findings and investigate the factors related to non-responder CP patients. Moreover, patients were followed for a short period, thus studies with long term follow-up should be encouraged to evaluate the persistence of rMV after effects.

## Conclusions

This study demonstrates that the treatment of drooling with rMV in children with CP is safe, well tolerated, and effective up to three months after the end of treatment. According to our findings, focal vibrations may improve swallowing mechanisms and favor the acquisition of the maturity of the oral motor control. Moreover, the reduction of the drooling improves the quality of life of the little patients and their caregivers.

## Figures and Tables

**Table 1:** Characteristics of the sample.

Participants	22
**Age (years)**	9,68 ± 3,45
**Affected side** Unilateral Bilateral	1 21
**Cerebral palsy subtype** Spastic Dyskinetic Ataxic	20 2 0
**Comorbid Factors** Epilepsy Intellectual disability	8 20
**Food intake** Unimpaired Dysphagic Gastronomy tube	4 17 1
**Speech** Unimpaired Dysarthric Anarthric	1 12 9

The quantitative variable age is expressed as mean ± standard deviation, whereas the qualitative variables as absolute frequencies.

**Table 2:** Median and IQR of evaluation at baseline (T0), post-treatment (T1) and two follow-up (T2-T3).

	Baseline (T0) Median (IQR)	Post-treatment (T1) Median (IQR)	Follow-up (T2) Median (IQR)	Follow-up (T3) Median (IQR)
DFSS	6.0 (6.0-7.0)	5.0 (4.0-6.0)	4.0 (4.0-5.0)	4.0 (4.0-5.0)
DIS	70.0 (57.1-78.0)	37.0 (23.9-47.2)	33.5 (18.5-55.0)	31.0 (18.8-54.5)
DQACT (%)	52.5 (40.0- 61.2)	25.0 (15.0-36.2)	20.0 (10.0-35.0)	10.0 (5.0-35.0)
DQREST (%)	42.5 (23.7-62.5)	22.5 (8.7-36.2)	15.0 (5.0-26.2)	10.0 (3.7-26.2)
VAS	75.0 (63.7-88.5)	36.5 (25.0-60.0)	30.0 (13.7-70.0)	30.0 (18.7-62.5)

**Table 3:** Friedman's test and Wilcoxon signed rank test (with Holm- Bonferroni sequential correction) results.

	Friedman test p-value	Wilcoxon signed-rank test					
		p-value T0-T1	p-value T0-T2	p-value T0-T3	p-value T1-T2	p-value T1-T3	p-value T2-T3
DFSS	<0.001	<0.001	<0.001	<0.001	0.090	0.112	0.999
DIS	<0.001	<0.001	<0.001	<0.001	0.666	0.808	0.808
DQACT (%)	<0.001	<0.001	<0.001	<0.001	0.234	0.168	0.234
DQREST (%)	<0.001	<0.001	<0.001	<0.001	0.204	0.340	0.647
VAS	<0.001	<0.001	<0.001	<0.001	0.999	0.999	0.999
